# Inverted Y-flap neovaginoplasty procedure combined with intracervical catheterization mold amnion graft in distal agenesis of vagina with transverse median vaginal septal: A case report

**DOI:** 10.1016/j.ijscr.2025.111204

**Published:** 2025-03-25

**Authors:** Roziana, Teuku Muhammad Yus, Misbahul Jannah, Anindyta Murfia Khairunnisa

**Affiliations:** a Department Obstetrics and Gynecology, Faculty of Medicine, Syiah Kuala University, Indonesia; b Department Radiology, Faculty of Medicine, Syiah Kuala University, Indonesia; c Medical School, Faculty of Medicine, Universitas Padjajaran, Indonesia

**Keywords:** Inverted Y-flap, Neovaginoplasty, Transverse vaginal septal, Vaginal agenesis

## Abstract

**Introduction and importance:**

Vaginal agenesis and transverse septa, rare Müllerian anomalies, make surgery challenging. The ideal neovaginoplasty with minimal complications remains uncertain. Hence, we report here an inverted Y-flap neovaginoplasty with an amnion graft for vaginal agenesis.

**Presentation of case:**

A 13-year-old girl with cyclic abdominal pain for a year was diagnosed with hematotrachelos, proximal hematocolpos, transverse vaginal septum, and a unicornuate uterus. She underwent Y-flap neovaginoplasty with an amnion graft after hysteroscopy. Discharged on day 5, she had an 8 cm vaginal length with no menstrual obstruction, showing excellent outcomes.

**Clinical discussion:**

The inverted Y-flap neovaginoplasty is a technique used in neovaginal reconstruction, which utilizes skin from the surrounding area to create a new vaginal cavity with an epithelial lining, maintaining its length. Neovaginoplasty with amnion grafts is a rewarding surgery, particularly in low-resource nations, because of its high success rate and low rates of postoperative discomfort, infection, and scarring.

**Conclusion:**

The inverted Y-flap neovaginoplasty with an amnion graft is a viable option for vaginal agenesis, showing success with minimal complications. Further research can refine surgical protocols.

## Highlights

• Vaginal agenesis and transverse vaginal septal are rare congenital conditions thus surgical management remains challenging in urogynecology
• The inverted Y-flap neovaginoplasty combined with intracervical catheterization mold amnion graft offers a feasible option for neovaginal reconstruction in vaginal agenesis patients
• Neovaginoplasty with amnion grafts is a rewarding surgery, particularly in low-resource nations, because of its high success rate and low rates of postoperative discomfort, infection, and scarring.

## 1 Introduction

Vaginal agenesis, a rare congenital condition, results from Müllerian duct malformation and affects 1 in 4000–5000 female births [1]. Vaginal agenesis presents as a missing vaginal canal, causing pain, amenorrhea, and marital consummation issues. It occurs in disorders like AIS (Androgen Insensitivity Syndrome) and MRKH (Mayer–Rokitansky–Küster–Hauser syndrome) [2,3]. Complete vaginal absence requires vaginoplasty to create a neovagina using various techniques [3,4]. Neovagina creation methods include skin grafts, bowel loops, and flaps like gluteal fold and DIEP (Deep Inferior Epigastric Flap) [5]. The inverted Y-flap uses local skin for neovagina creation, ensuring proper length with high success and minimal complications [6].

Maintaining patency in neovaginoplasty is challenging due to natural wound healing. Using a stent or mold prevents stenosis, secures grafts, and promotes epithelization [3,4]. The right vaginal mold is key to neovaginal reconstruction, but data on mold types and outcomes is limited [3]. The amniotic membrane aids epithelization, making it ideal for grafting in neovaginal reconstruction [4]. Gari et al. [13] retrospective analysis of 10 patients found 100 % anatomical and 80 % functional success, with no major complications in neoconstruction of vagina using amnion graft. We report an inverted Y-flap neovaginoplasty procedure combined with an intracervical catheterization mold amnion graft following hysteroscopy diagnostics in a 13-year-old girl with distal agenesis of vagina and transverse median vaginal septal. This case report has been reported in line with the SCARE Criteria [7].

## 2 Patient information

**Fig. 1:**
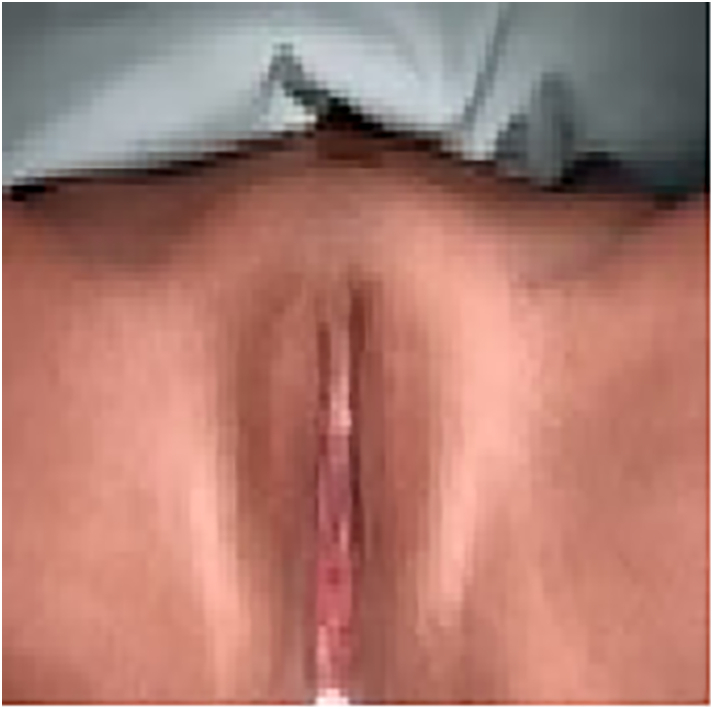
Pre-operative: no introitus of vagina, mullerian vestigate +/+.

A 13-year-old girl was admitted with cyclic abdominal pain and abdominal enlargement for a year. She had primary amenorrhea due to the absence of a vaginal canal, similar to her younger sister. Her secondary sexual characteristics were normal (Tanner stage IV). Examination revealed a mildly distended abdomen with a 3 × 3 cm palpable, slightly tender intra-abdominal mass and positive peristalsis. On the gynecology examination we found no introitus of the vagina, mullerian vestigate +/+(Fig. 1), Rectal examination showed a smooth anal mucosa, a 5 × 5 cm anterior mass with limited mobility and pain, and a palpable cervix.

## 3 Diagnostic assessment

**Fig. 2:**
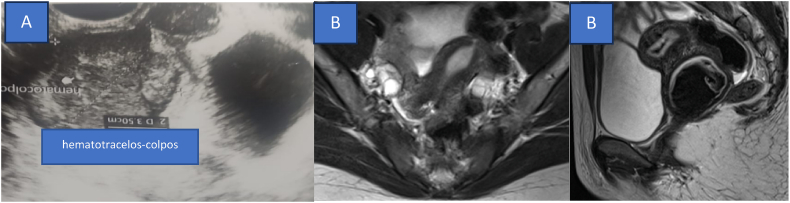
(A). Abdominal ultrasonography showing echo intensity in cervix suggested hematocolpos. (B) MRI showing hyperintense cystic lesions in T1W1 and hypointense in T2W1 and vaginal distal was not visible agenesis distal vaginal with hematocolpos.

Transrectal ultrasound showed an anteflexed uterus with a “blades appearance” (6.76 × 3.03 × 1.1 cm) and no fluid or mass. The right and left ovaries measured 1.99 × 1.08 cm and 1.29 × 1.36 cm, respectively. A 4.98 × 4.16 cm hypoechoic mass was detected inferior to the uterus, consistent with proximal hematocolpos and hematotrachelos (Fig. 2a). MRI confirmed findings consistent with hematocolpos (Fig. 2b). The patient was diagnosed with abdominal pain due to hematotrachelos, proximal hematocolpos, distal vaginal agenesis, a transverse vaginal septum, and a unicornuate uterus.

## 4 Therapeutic intervention

**Fig. 3:**
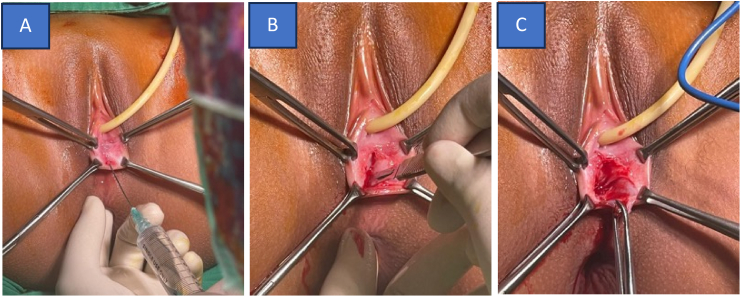
Intraoperative. (A) infiltration with diluted epinephrine. (B) inverted Y-Flap incision was made. (C) continue dissection to separate tissues.

**Fig. 4:**
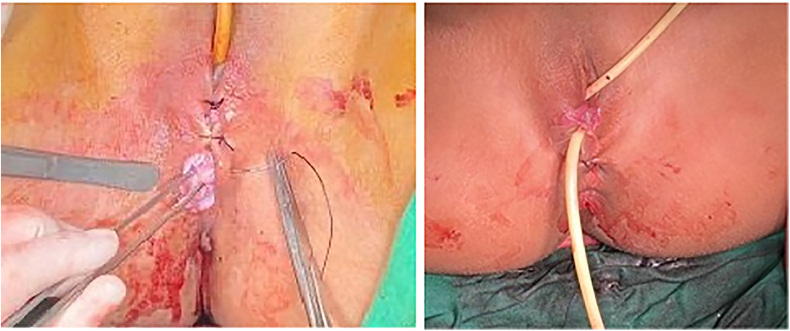
Surgical incision was closed and post-operative appearance.

**Fig. 5:**
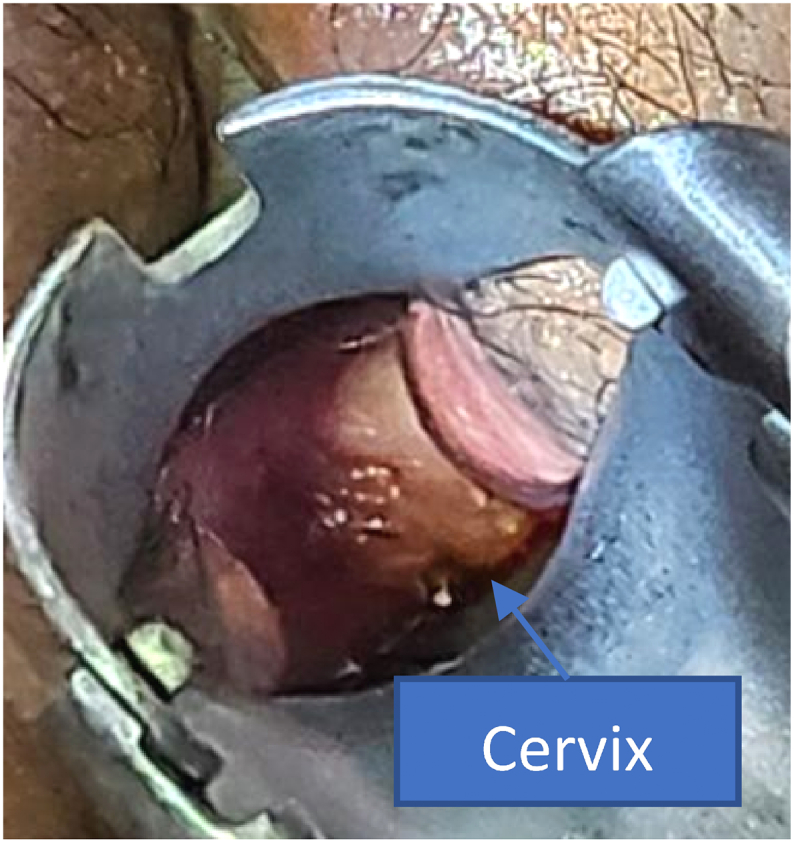
Follow up (vaginal examination 2 months after surgery).

We reconstructed a neovagina using the inverted Y-Flap technique and an intracervical catheterization mold amnion graft after hysteroscopic evaluation. The procedure was done with the patient in the lithotomy position under spinal anesthesia. A Foley catheter was placed, and Ellis clamps were applied bilaterally to the Müllerian vestige and fourchette (Fig. 3). Diluted epinephrine was injected, and an inverted Y-shaped incision was made. Dissection extended to the proximal vagina, guided by abdominal ultrasound and rectal examination. As 10 mL of fluid was injected, 100 mL of dark red menstrual blood drained from the introitus. A 0.3 × 0.3 cm vaginal septum, located 2 cm from the introitus, was excised and sent for histopathology. Layer-by-layer dissection continued until the cervix was exposed, revealing an 8 cm TVL (Total Vaginal Length), elongated due to hematocolpos (Fig. 4). Hysteroscopy revealed a left-elongated uterus with a visible left tubal isthmus, while the right was absent, indicating a unicornuate unicollis uterus. Uterine sondage measured 7 cm in the left hemiuterus. The incision was closed with PGA (PolyGlycolic Acid) No.2, bleeding was controlled, and a mold amnion graft with an intracervical catheter was placed. A catheter was installed into the introitus vagina and fixed on the labia using Silk No.2 then maintained for 7 days. The surgery lasted 60 min with 100 cc of blood loss and 100 cc of urine output (Fig. 5).

## 5 Follow up and outcome

The patient was discharged on the 5th day. A total vaginal length of 8 cm without obstruction of menstrual flow, the follow-up findings were outstanding. The patient 2 months after surgery follow up result showed in Fig. 5.

## 6 Discussion

A 13-year-old girl was admitted with cyclic abdominal pain and an enlarged abdomen for a year due to vaginal agenesis. She had normal secondary sexual characteristics (Tanner stage IV). Vaginal agenesis, often caused by MRKH syndrome, leads to symptoms like lower abdominal pain, amenorrhea, and infertility. MRKH, a Müllerian anomaly causing uterovaginal malformation, is typically diagnosed during primary amenorrhea evaluation and affects 1 in 5000 females [8]. Individuals with MRKH syndrome develop normal secondary sexual characteristics due to a typical female karyotype and functional ovaries. While 7–10 % have a functional endometrium, most have a rudimentary uterus [2,9].

Transverse vaginal septum (TVS), a type of Müllerian anomaly, occurs in 1 in 70,000 cases. It can appear at any vaginal level, most often in the upper or middle third. Treatment typically involves surgical opening, using either a simple incision or skin grafting, sometimes incorporating the septum itself, such as in Y-plasty [10].

We performed neovagina reconstruction using an inverted Y-Flap. Various surgical methods exist, including flaps, bowel loops, and skin grafting. In the 1930s, McIndoe achieved success with split-thickness skin grafts (STSG) inverted over a stent [5]. Full-thickness skin grafts (FTSG) contract less than STSG during healing. Moreover, Skin grafts provide a single-stage procedure for a hairless neovagina with adequate depth and width and low postoperative risks. However, they may cause prolapse, scarring, graft shrinkage, HPV-related issues, poor sensation, and lack of lubrication [11]. Common flaps for vaginal agenesis include gluteal fold, DIEP, and other skin flaps [5]. Flap techniques offer better depth, less contraction, and shorter dilation needs but come with donor site scarring, procedural complexity, bulkiness, and no self-lubrication [11]. The inverted Y-flap is a neovaginoplasty technique for MRKH and AIS patients, using local skin to maintain vaginal length. A five-year study in Bandung, Indonesia, found it successful with no major complications, strictures, or contractures. Patients also reported pain-free intercourse, highlighting its functional and aesthetic benefits [6]. Ulaganathan compared three vaginal construction techniques for vaginal agenesis and concluded that for patients with inconsistent follow-ups and stent use, the flap procedure, which has the lowest contraction rate, is the preferable option [12].

After neovaginal construction, an amnion graft with an intracervical catheter mold maintains patency and prevents stenosis. Amniotic membranes, widely used in medicine, support healing with anti-inflammatory and anti-scarring properties. They are affordable, accessible, and non-immunogenic due to the absence of HLA antigens [9]. Amnion grafts in neovaginoplasty, especially for MRKH, offer high success with minimal discomfort, infection, and scarring, making them ideal for low-resource settings [9]. Our patient was discharged on day 5 with an 8 cm vaginal length and no menstrual obstruction. Studies support amnion grafts for neovaginoplasty, with a 50-patient MRKH study showing an 8.2 cm mean vaginal length and a 30.8 FSFI score, indicating good outcomes [14]. A study reported 100 % anatomical success, a 5.5 cm mean vaginal length, and 80 % functional success per the FSFI (Female Sexual Function Index) [13]. Amnion grafts are safe with minimal complications. In a study of 28 patients, only one had major perioperative issues, with most achieving satisfactory results [15]. A study found no major complications, like pelvic abscesses or chronic discharge, confirming its safety [13].

This article adds insight into the use of amnion grafts that can be used in situations with limited resources, but in this case, follow-up was only carried out up to 2 months after surgery, so monitoring of complications and surgical results was less than optimal, which is a weakness of this article.

## 7 Conclusion

The inverted Y-flap neovaginoplasty with an amnion graft is a safe, effective option for vaginal agenesis. Further research can refine techniques and outcomes.

## CRediT authorship contribution statement

**Roziana**: concept, operator, data analysis, drafting and revising, final approval.

**Teuku Muhammad Yus**: data analysis, drafting and revising.

**Misbahul Jannah**: data collection, data analysis, writing the paper.

**Anindyta Murfia Khairunnisa**: Writing the paper, drafting and revising.

## Consent

Written informed consent was obtained from the patient's parents/legal guardian for publication and any accompanying images. A copy of the written consent is available for review by the Editor-in-Chief of this journal on request.

## Ethical approval

Because this is the case report and it is not a research, ethical approval was not required.

## Guarantor

Roziana acts as the author and the guarantor of this article. Author is fully responsible for the work, the conduct of the study, data accessibility and decision to publish.

## Provenance and peer review

Not commissioned, externally peer-reviewed.

## Funding

Source of funding is form the author only. There is no involvement of sponsor.

## Registration of research studies

N/A.

## Declaration of competing interest

This case report do not have any relationship with other people or organizations.

## Data Availability

All data and tables used to support the findings of this study are included within the article and available upon request to the corresponding author.
